# Blood neutrophil counts are associated with exacerbation frequency and mortality in COPD

**DOI:** 10.1186/s12931-020-01436-7

**Published:** 2020-07-01

**Authors:** Mike Lonergan, Alison J. Dicker, Megan L. Crichton, Holly R. Keir, Melissa K. Van Dyke, Hana Mullerova, Bruce E. Miller, Ruth Tal-Singer, James D. Chalmers

**Affiliations:** 1 Scottish Centre for Respiratory Research, University of Dundee, Ninewells Hospital and Medical School, Dundee, DD1 9SY UK; 2 Epidemiology, Value Evidence and Outcomes, GSK R&D, Collegeville, PA USA; 3 Epidemiology, Value Evidence and Outcomes, GSK R&D, Uxbridge, UK; 4 Medical Innovation, Value Evidence and Outcomes, GSK R&D, Collegeville, PA USA

**Keywords:** COPD, Mortality, Eosinophil, Neutrophil, Microbiome

## Abstract

**Background:**

Identifying patients with COPD at increased risk of poor outcomes is challenging due to disease heterogeneity. Potential biomarkers need to be readily available in real-life clinical practice. Blood eosinophil counts are widely studied but few studies have examined the prognostic value of blood neutrophil counts (BNC).

**Methods:**

In a large population-based COPD registry in the East of Scotland (TARDIS: Tayside Allergic and Respiratory Disease Information System), BNC were compared to measures of disease severity and mortality for up to 15 years follow-up. Potential mechanisms of disease modification by BNC were explored in a nested microbiome substudy.

**Results:**

178,120 neutrophil counts were obtained from 7220 people (mean follow up 9 years) during stable disease periods. Median BNC was 5200cells/μL (IQR 4000-7000cells/μL). Mortality rates among the 34% of patients with elevated BNCs (defined as 6000-15000cells/μL) at the study start were 80% higher (14.0/100 person years v 7.8/100py, *P* < 0.001) than those with BNC in the normal range (2000-6000cells/μL). People with elevated BNC were more likely to be classified as GOLD D (46% v 33% *P* < 0.001), have more exacerbations (mean 2.3 v 1.3/year, P < 0.001), and were more likely to have severe exacerbations (13% vs. 5%, P < 0.001) in the following year. Eosinophil counts were much less predictive of these outcomes. In a sub-cohort (*N* = 276), patients with elevated BNC had increased relative abundance of Proteobacteria and reduced microbiome diversity.

**Conclusion:**

High BNC may provide a useful indicator of risk of exacerbations and mortality in COPD patients.

## Background

Biomarkers are needed in Chronic Obstructive Pulmonary Disease (COPD) for effective risk stratification and to guide personalized treatment. Blood eosinophil counts have been extensively investigated to stratify patients with COPD, based on evidence that blood and sputum eosinophil counts are linked, and both are associated with increased exacerbation frequency, reduced lung function and responsiveness to inhaled corticosteroids (ICS) [1]. Only a minority of COPD patients, however, exhibit eosinophilia; COPD is predominantly characterized as a neutrophilic inflammatory disorder [2, 3]; because the most abundant inflammatory cell in blood and sputum is the neutrophil, and because neutrophil proteases can recapitulate many of the features of COPD in disease models, such as emphysema and mucus hypersecretion [4–6]. The introduction of blood eosinophil counts into clinical practice recommendations from GOLD illustrates the potential for simple clinical measures to come into daily practice, but whether blood neutrophil counts (BNC) can be used to inform clinical decisions has not been extensively investigated in large cohorts.

Increased peripheral neutrophil counts are a reflection of systemic inflammation which is linked to disease severity and co-morbidities in COPD. Correlations between disease severity stage and neutrophil activation markers (e.g. neutrophil elastase (NE), myeloperoxidase and neutrophil extracellular traps) in sputum and bronchoalveolar lavage have been shown previously [7–9].

We therefore hypothesized that BNC would be a predictor of COPD severity, exacerbation and mortality.

## Methods

### Study design

Seven thousand two hundred twenty people with a COPD diagnosis were identified within the TARDIS (Tayside Allergy and Respiratory Disease Information System) database. Details of TARDIS have been previously described and are detailed online [10]. In brief, since 2008 patients age > 40 years, with a post-bronchodilator FEV_**1**_/FVC ratio < 0.7 (70%) and a clinical diagnosis of COPD were invited to participate in a structured primary care cohort. Patients were reviewed annually for spirometry, recording of symptoms and exacerbation history. Patient’s TARDIS data was linked to regional prescribing databases, and databases that record hospitalizations and deaths nationally. The study was approved by the local research ethics committee approval number 13/ES/0030.

### Blood neutrophil counts

The index date for each participant was set as the date of the first stable BNC recorded after they had been in the TARDIS study for 12 months. A stable BNC was one considered representative of times when that person’s COPD was stable and unaffected by exacerbations or short-tern medical treatment. BNCs made between 1 week before and 4 weeks after the start of a COPD exacerbation, defined as either receipt of a short course of prednisolone (defined as an acute prescription for at least 30 mg/day for at least 5 days) or a respiratory hospitalization, were excluded as potentially being elevated by the immediate effects of the exacerbation or associated corticosteroid treatment. The online supplementary material gives details of the derivation of each variable used.

Extremely low and extremely high BNCs can result from various diseases and have the potential to dominate analyses that dichotomize or treat effects as linear. Therefore, the BNCs were split into four categories: low (<2000cells/μL); normal (2000–6000cells/μL); elevated (6000–15,000cells/μL); and extreme (> 15,000cells/μL). The upper bound of the normal range for BNC has often been taken to be 8000cells/μL in clinical practice so the models were refitted using that cut-off point in a sensitivity analysis. Models were also fitted, including all individuals, with BNC as a linear term as a sensitivity analysis. The primary interest was how people with elevated BNC differed from those in the normal range.

### Microbiome sub cohort

Two hundred seventy-three patients in the TARDIS cohort had been previously recruited into prospective longitudinal studies as previously described [10]. Participants were clinically assessed after giving written informed consent (Relevant medical history, Spirometry, St Georges Respiratory Questionnaire (SGRQ), GOLD 2017 scores, COPD Assessment Test (CAT), and Medical Research Council (MRC) Dyspnoea Score), and sputum and blood obtained when clinically stable at study enrolment. Full blood counts and C-reactive protein were measured at every visit. DNA was extracted from the induced sputum obtained at visits and 16S rRNA microbiome sequencing performed as detailed in the online supplement. All sequences generated are available in the NCBI Sequence Read Archive under the Bioproject accession numbers PRJNA316126 and PRJNA539959.

### Validation in ECLIPSE

BNCs were measured as a biomarker in the ECLIPSE cohort, a longitudinal COPD cohort study that has been previously described [11]. The original study had a duration of 3 years. Patients who provided consent for additional follow-up to 8 years were included and the relationship between baseline BNCs and survival was examined after adjustment for confounders using Cox proportional hazards regression.

### Statistical analysis

Data were summarized by BNC group to give simple comparisons. Regression models were then used to look at how other potential explanatory variables might change the results. Cox proportional hazard models of all-cause mortality were fitted, along with survival models of mortality recorded as being due to ICD10 code J44 (COPD) and ICD10 codes J00–99 (all respiratory mortality). The models of disease specific mortality treated other causes of mortality as competing risks. Generalized linear models (GLMs), with log links and negative binomial errors, were used to look at the frequency of severe exacerbations (defined as hospitalization with the main condition recorded as ICD10 code J44), and all acute exacerbations (defined as respiratory hospitalization or receiving a course of prednisolone), in the 12 months from the index date.

To examine changes in stable FEV_1_, mixed models were fitted. These contained an intercept and slope for each BNC group as fixed effects, along with an intercept and slope for each individual as random effects. Because the mixed model assumes linear changes in FEV_1_, and there was high mortality in this cohort, a second set of mixed models was fitted using only measurements taken within 3 years of the index dates.

Unadjusted models were fitted, using the four BNC levels to predict the outcomes, comparing the other groups to the normal BNC group. The models were then adjusted for age, sex and smoking status and refitted. To examine the stability of the results, 35 further versions of each model were fitted, each adjusting for another plausible confounding variable as listed in e-Table 1.

## Results

**Fig. 1:**
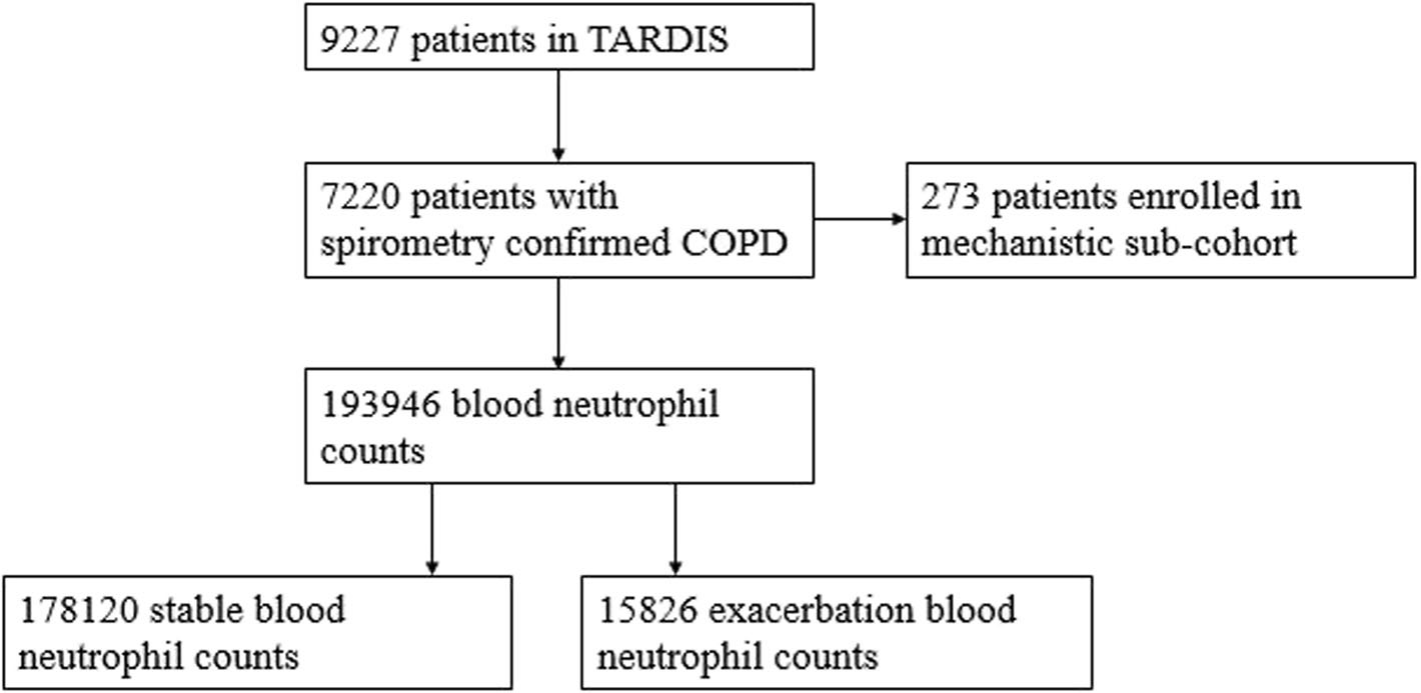
Flow chart of numbers of patients and samples in TARDIS, and each set of analyses

From the 7220 COPD patients in TARDIS, 178120 stable and 15,826 exacerbation associated BNCs were obtained (Fig. 1); their clinical characteristics at index are shown in Table 1. The median age of patients at index was 75 years (interquartile range (IQR) 65–81), 48% were male, 4525 (63%) were ICS users and 3494 (49%) were current smokers whilst median FEV_1_ was 1.6 L (IQR 1.1–2.0). Comparing the normal and elevated groups, the elevated group were older, more likely to be smokers, receiving ICS, and had more exacerbations and a lower index FEV_1_ (Table 1). 2% of stable BNCs were classed as low; 50% as normal, 43% as elevated and 4% as extreme. Less than 1% of the exacerbation BNCs were classed as low; 38% as normal, 56% as elevated and 6% extreme.

**Table 1:** Characteristics of the cohort at index date

	All	Normal index BNC	Elevated index BNC	Significance
n	7220	4465 (62%)	2462 (34%)	
Age	75 (65, 81)	72 (64, 80)	74 (67, 82)	*P* < 0.001
Male Gender	3470 (48%)	2149 (48%)	1171 (48%)	*P* = 0.67
BMI	28 (24, 31)	28 (24, 31)	28 (23, 32)	*P* = 0.63
ICS use^a^	4525 (63%)	2680 (60%)	1635 (66%)	*P* < 0.001
LABA only	146 (2%)	101 (2%)	42 (2%)	*P* = 0.14
LAMA only	555 (8%)	339 (8%)	196 (8%)	*P* = 0.61
LABA & LAMA	168 (2%)	103 (2%)	58 (2%)	*P* = 0.96
SABA	6068 (84%)	3672 (82%)	2137 (87%)	*P* < 0.001
Current smokers	3494 (48%)	2104 (47%)	1264 (51%)	*P* = 0.02
Ex-smokers	3300(46%)	2071 (46%)	1078 (44%)	
Spirometry				*P* < 0.001
FEV_1_ (Litres)	1.58 (1.11, 1.98)	1.64 (1.23, 1.91)	1.49 (1.00, 1.87)	
GOLD 2017				*P* = 0.002
A	332 (8%)	243 (9%)	82 (6%)	
B	2174 (51%)	1464 (54%)	646 (46%)	
C	132 (3%)	99 (4%)	30 (2%)	
D	1620 (38%)	888 (33%)	634 (46%)	
Index total white cell count (cells/μL)	8300 (6900, 1030)	7300 (6300, 8300)	10,800 (9700, 12,600)	*P* < 0.001
Index BNC (cells/μL)	5200 (4000, 7000)	4300 (3600, 5100)	7600 (6600, 9400)	*P* < 0.001
Index eosinophil count (cells/μL)	180 (90, 290)	200 (120, 300)	150 (60, 280)	*P* < 0.001
< 150	2960 (41%)	1518 (34%)	1206 (49%)	
> 150; < 300	2521 (35%)	1765 (40%)	720 (30%)	
> 300; < 500	1253 (17%)	869 (19%)	369 (15%)	
> 500	486 (7%)	313 (7%)	167 (7%)	
Exacerbation frequency (year to index date)				*P* < 0.001
0	2679 (37%)	1797 (40%)	792 (32%)	
1	1653 (23%)	1075 (24%)	535 (22%)	
2	1033 (14%)	606 (14%)	380 (15%)	
3 or more	1855 (26%)	987 (22%)	755 (31%)	
Severe exacerbation (year to index date)	577 (8%)	162 (4%)	334 (14%)	
Exacerbation frequency (year from index date)				*P* < 0.001
0	2989 (41%)	1870 (42%)	988 (40%)	
1	1549 (21%)	1019 (23%)	493 (20%)	
2	1005 (14%)	649 (15%)	329 (13%)	
3 or more	1677 (23%)	927 (21%)	652 (26%)	
Severe exacerbation (year from index date)	633 (9%)	245 (5%)	322 (13%)	
Crude mortality rate (per 100 person-years)	10.0	7.9	14.0	*P* < 0.001

Abbreviations: *BNC* Blood Neutrophil Count, *ICS* Inhaled corticosteroids, *BMI* Body mass index, *MRC* Medical Research Council, *FEV*_*1*_ Forced expiratory volume in 1 s, *FVC* Forced vital capacity, ^a^includes medication used in combination with other bronchodilators or inhaled steroids. Each cell contains the median (IQR) or n (%). Not every measurement is available for every individual at the index visit hence not all percentages add up to 100%. T-tests were used to estimate the significance of differences between the characteristics of the groups, except for categorical variables where Chi-squared tests were used and ones where the distributions within either group failed the Shapiro-Wilks test of normality and the Mann-Whitney test was used instead. The large sample size made some small differences statistically significant.

### Blood eosinophil analysis

**Fig. 2:**
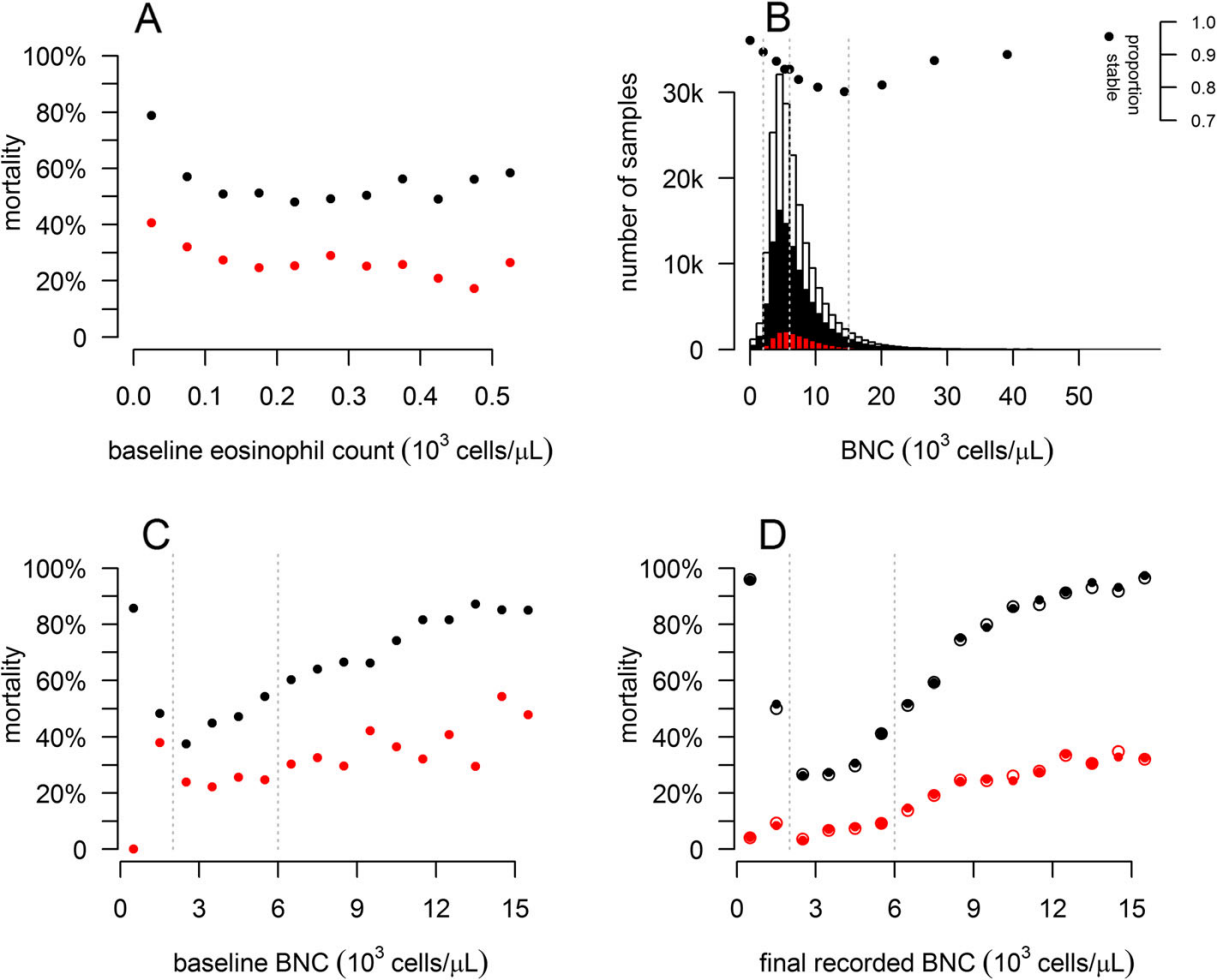
**a:** The proportion of individuals recorded as having died at the end of follow up (black) plotted against their index blood eosinophil count. The red symbols are proportions (out of all individuals) with death from a cause recorded as falling within ICD10 category J44 (COPD). **b:** Distribution of blood neutrophil counts (BNCs) showing all values (hollow bars); all measurements from individuals with stable and exacerbating results available (black bars) and measurements made during exacerbations (red bars). The black dots show the proportion of BNCs made outside of exacerbation periods. Three vertical dotted lines indicate the cut-offs applied to separate BNCs into normal (<6000cells/μL), elevated (6000–15,000cells/μL) and extreme (> 15,000cells/μL) **c:** The proportion of individuals recorded as having died by the end of follow up (black) plotted against their index BNC. The red symbols are proportions (out of all individuals) with death from a cause recorded as falling within ICD10 category J44. Three vertical dotted lines indicate the cut-offs applied to separate BNCs into low (<2000cells/μL), normal (2000–6000cells/μL) and elevated 6000–15,000cells/μL) **d:** as C, but using the last BNC recorded for each individual. Solid points are based on final stable BNC, while hollow ones include BNC recorded during exacerbations

The association between index blood eosinophil counts and mortality was weak and only evident in patients with blood eosinophil counts <100cells/μL (Fig. 2a). No significant difference in all-cause mortality, mortality due to COPD or change in FEV_1_ was observed between the different blood eosinophil count groups. The total exacerbation rates were also indistinguishable, though the rate of severe exacerbations was lower among those with elevated index blood eosinophils than in all other groups (e-Fig. 1 and e-Table 2).

### Elevated BNCs and increased mortality

**Fig. 3:**
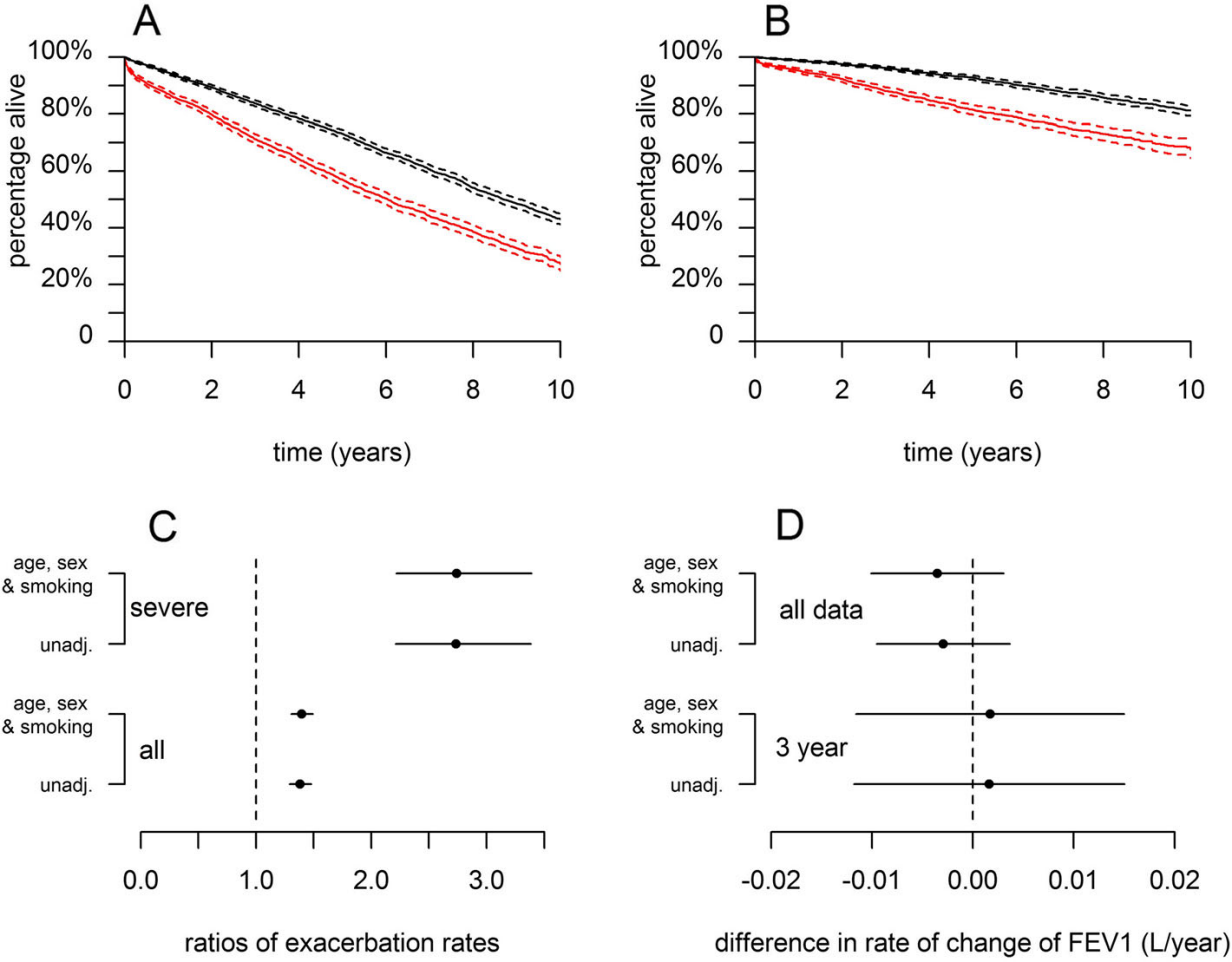
**a**: Kaplan Meier survival curves, with 95% confidence intervals, showing all-cause mortality for the normal (black) and elevated (red) BNC groups adjusted for age, sex, and smoking status. **b**: As **A**, but representing mortality recorded as ICD10 J44, with all other mortality as a competing risk. **c**: Ratios of the numbers of all, and severe, exacerbations over the year from the index date from both unadjusted models and models adjusted for age, sex and smoking status. All are relative to the normal BNC group. **d:** Rates of change of FEV_1_, estimated by fitting mixed models to either all data or only that obtained in the three years from each individual’s index date. For clarity, the low and extreme BNC groups are not shown here, though equivalent plots showing all four BNC groups are shown in supplementary e-Figure 6

BNC were significantly higher during exacerbations (*P* < 0.001; Fig. 2b), though this difference was small compared to the within-individual variability (median coefficient of variation 0.36; IQR 0.26–0.48; e-Figure 2). Despite this variability, a pattern of mortality risk being higher for those with BNC outside the normal range, was evident. Unsurprisingly, the association of mortality with index BNC (Fig. 2c) was less strong than that for the final BNC (Fig. 2d). These patterns were similar for all-cause mortality and that recorded as due to COPD. The patterns in the raw data (Fig. 2c & d) remained as statistically significant differences (*P* < 0.001) in survival between the BNC groups even after adjustment for age, sex and smoking history, with 10 year survival for those with an elevated BNC being about 2/3 of that for those with normal BNC at index (Fig. 3a and b). Very similar results were obtained for unadjusted models, and those considering all respiratory (ICD code J) deaths (e-Figure 3). The adjusted model with BNC as a linear term estimated the hazard ratio for all-cause mortality at 1.067 (95%CI: 1.060–1.073 per 1000cells/μL), and that for COPD mortality at 1.091 (95%CI: 1.082–1.100). These estimates, while still significant, are lower than the between group differences (e-Table 3) because there is high mortality among the group with low index BNC. Additional models did not identify any important confounding effects (e-Figures 4 and 5).

The elevated BNC group had more exacerbations than the normal BNC group in both the year before index (mean 2.3 vs 1.3 exacerbations/year, *P* < 0.001) and the year following index (1.9 vs 1.5, *P* < 0.001). The incident rate ratios for all exacerbations or severe exacerbations alone in the 12 months following index date were determined, with the normal BNC group as reference; two models are shown (Fig. 3c), one unadjusted and the second adjusted for age, sex and smoking history. The adjusted models indicated that, for patients with an elevated BNC, there was a 40% increase in total exacerbations, and a 170% increase in severe exacerbations in the year from index relative to the rates for those with normal BNC (*P* < 0.001 in both cases). The adjusted model with BNC as a linear term, estimated that each increase of 1000cells/μL in BNC was associated with the total exacerbation rate increasing by a factor of 1.05 (95%CI: 1.04–1.06). For severe exacerbations, the figure was 1.17 (95%CI: 1.14–1.20). In the year from index date the much smaller group of patients with an extreme BNC had an increase in all and severe exacerbations of 96 and 450% (*P* < 0.001 in both cases; e-Figure 6).

Additional GLMs did not identify any important confounding effects (e-Figures 7 and 8). When looking at all exacerbations, the neutrophil effect remained significant in every case, with only the total number of exacerbations in the year up to index date attenuating the magnitude of the effect. (e-Figure 7). Restricting the analysis to the younger half of the cohort did not change the results (e-Figure 9). Changing the upper limit of the normal range from 6000 to 8000cells/μL halved the size of the elevated BNC group, and widened confidence intervals, without changing the overall results or conclusions (e-Figure 10).

The overall rate of change in FEV_1_, across the whole cohort was − 0.038 (95%CI: − 0.034, − 0.041) L/yr or 38 mL/yr. The rate within the elevated BNC group was indistinguishable from the normal BNC group (Fig. 3d). While estimated rate of decline for the elevated BNC group was slightly faster (difference − 0.004; 95%CI: − 0.010, 0.003), the difference was not statistically significant, and was reversed when consideration was restricted to only the first 3 years after the index date (difference between groups 0.002; 95%CI: − 0.012, 0.015). However, these confidence intervals are too wide to entirely rule out the existence of a clinically meaningful difference.

### Microbiome sub-cohort

**Fig. 4:**
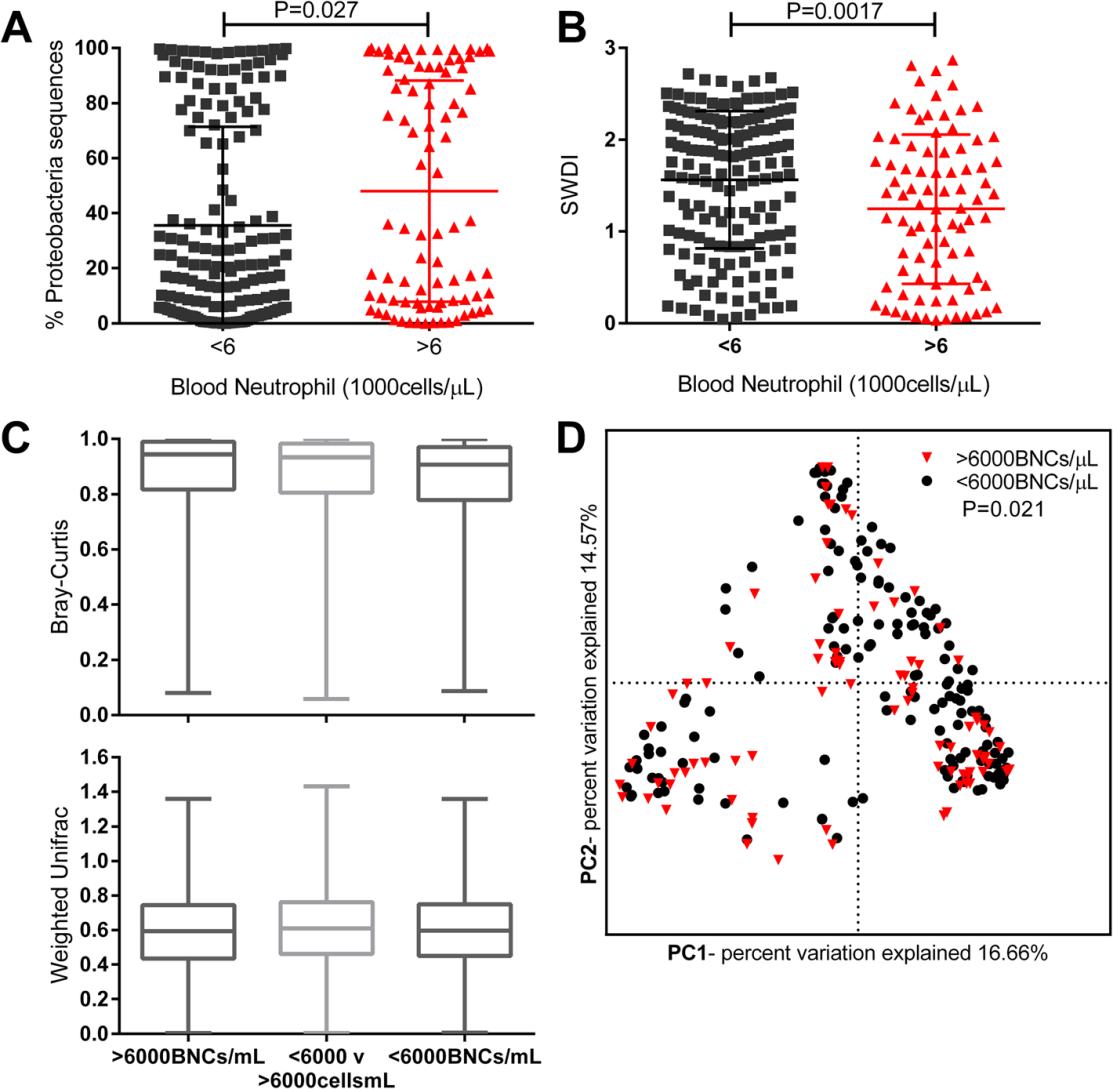
Comparisons of BNCs to **a:** percentage Proteobacteria in the microbiome from *n* = 246 stable COPD patients. **b:** SWDI, a measure of the microbiome diversity. **c:** Bray Curtis dissimilarity index and Weighted UNIFRAC were used to assess the differences in the microbiome between pairs of patients with the same BNC (<6000BNCs/μL or > 6000BNCs/μL) and between those pairs of patients with different BNCs (<6000BNCs/μL v > 6000BNCs/μL). **d:** PCoA plot showing the relationship between samples according to BNC

Two hundred seventy-three patients provided at least one sputum sample, when clinically stable, of which 246 samples passed sequencing quality control to be included in the analysis. Utilizing the same cut offs as the main TARDIS cohort, 1 patient had a low BNC, 159 were normal, 79 elevated and 7 extreme. For the purpose of the microbiome analysis, low and normal patients were grouped as <6000cells/μL, elevated and extreme as >6000cells/μL. At the phylum level, the microbiome was dominated by Proteobacteria and Firmicutes, corresponding to dominant genera of *Haemophilus* (*n* = 59 patients) and *Streptococcus* (*n* = 78) at the genus level (e-Figure 11). Additional dominant genera observed included: *Veillonella* (*n* = 55), *Neisseria* (*n* = 12), *Pseudomonas* (*n* = 11), *Moraxella* (*n* = 8) and *Prevotella* (*n* = 7). The BNC count of the samples was significantly associated with increased % Proteobacteria (*P* = 0.027) and reduced Shannon Wiener Diversity Index (*P* = 0.0017) (Fig. 4 a and b). Beta diversity was assessed using the Bray Curtis Dissimilarity Index and Weighted UNIFRAC (Fig. 4c). Between patients, the microbiome composition was shown to be significantly different by PERMANOVA when comparing patients with BNCs <6000cells/μL to patients with BNCs >6000cells/μL (*P* = 0.014), which remained statistically significant (*P* = 0.021) when adjusted for age, sex and smoking status (Fig. 4d).

### ECLIPSE validation cohort

The observation that BNC was associated with mortality in COPD was confirmed in the ECLIPSE cohort. From 2164 COPD patients enrolled in ECLIPSE, 1934 participants were alive at the end of the original 3 year study period. One thousand five hundred fifty-five participants consented to an assessment of survival at 8 years; the 379 patients who did not participate in the 8 year survival assessment were censored. In the Cox proportional hazards model adjusted for age, prior hospitalization and BODE index, BNC was independently associated with decreased survival as a linear variable (HR 1.20 95%CI 1.11–1.29, *P* < 0.0001).

## Discussion

This study shows that BNCs are strongly related to long term mortality in COPD and to the frequency of exacerbations and hospitalizations. Patients with BNCs >6000cells/μL had markedly poorer outcomes regardless of the time point in their COPD disease course that the BNC was measured. We evaluated BNCs 12 months after initial enrolment into the TARDIS, which would be close to diagnosis for many patients, and the penultimate BNC in the study which was later in the disease course; in both cases BNC was strongly and independently associated with poor outcomes. Obtaining a similar result at two different time points represents a degree of internal validation and the finding was externally validated in the ECLIPSE cohort. In ECLIPSE, increasing BNC was clearly associated with increased mortality even after adjustment for the BODE index. In the TARDIS cohort, both all causes and respiratory mortality were increased, and this is consistent with the knowledge that systemic inflammation contributes to both respiratory morbidity and co-morbidities, which are a major feature of COPD [12, 13].

We extended our findings by demonstrating a statistically significant difference in the microbiome between patients with different stable BNCs. Patients with higher blood neutrophil counts had a lower microbiota diversity, a parameter known to be associated with more severe COPD and a higher frequency of exacerbations, and also had a higher relative abundance of Proteobacteria [14]. This phylum includes the pathogenic genera *Haemophilus*, *Moraxella* and *Pseudomonas* which are linked to lung neutrophilic inflammation and poorer clinical outcomes [15]. It is not possible to determine whether differences in the microbiome are directly causing increased blood neutrophil counts or if both are a reflection of a separate process.

The main role of neutrophils is to kill bacteria, principally through phagocytosis. Neutrophils migrate from the peripheral blood circulation towards inflammatory stimuli due to chemotactic factors, becoming activated and releasing NE as they migrate through the extracellular matrix [2]. Killing of bacteria is achieved either through phagocytosis, by releasing reactive oxygen species (ROS), lactoferrin and proteinases, or by producing NETs [3, 16]. However, in COPD, despite the presence of large numbers of neutrophils in the lungs, disease progression continues, driven by exacerbations which are often infectious in nature [17].

It appears that the large numbers of neutrophils observed both in the lungs and in systemic circulation are defective in their innate immune function: Ex vivo studies have shown that neutrophils from COPD patients are more activated; demonstrating increased levels of chemotaxis but that the accuracy of the direction of movement is impaired [18–20]. Milara et al, in a study on peripheral blood neutrophils from early onset COPD patients, showed the NE release was 2-fold greater and ROS release was 30% greater compared to healthy controls [18]. Jones et al compared COPD patients with or without a frequent exacerbator phenotype; they observed that bacterial stimulated neutrophil degranulation was greater in the frequent exacerbator group, but that all COPD derived blood neutrophils had a blunted fMLP stimulated oxidative burst response compared to healthy controls [21].

The effects of COPD are not restricted to the lungs; evidence of persistent systemic inflammation has been assessed by measuring white blood count, C-reactive protein, interleukins 6 and 8 or TNF-α in serum [8, 22]; 16% of COPD patients in the ECLIPSE cohort were shown to have markers of systemic inflammation present [23, 24]. Wouters et al showed systemic markers of inflammation were upregulated during exacerbations [9, 25, 26]. Comorbidities (e.g. osteoporosis, hypertension, cardiovascular disease or diabetes) are commonly present in COPD patients [12, 13], with more comorbidities being recorded in patients with the most severe lung function restriction in the ARIC and CHS studies [27]. In the BODE cohort, COPD patients had an average of four co-morbidities [28], with comorbidities being the primary cause of death in 60% of non-survivors in two random controlled trials [29]. Our study adds to this evidence that COPD patients have persistent systemic inflammation and that this is linked to poor clinical outcomes.

How might BNCs be useful in clinical assessment of patients with COPD? Unlike blood eosinophil counts there is not yet evidence that BNCs can help guide therapy. Unfortunately, to date, there are few effective treatments for neutrophilic inflammation since NE inhibitors, CXCR2 antagonists and other neutrophil specific therapies have not yet been shown to improve outcomes in broad populations of COPD patients [30, 31]. Nevertheless, there is an established precedent for blood biomarkers being used to guide treatment of inflammatory endotypes, as in the case of blood eosinophil counts and anti-IL-5 therapy. It is theoretically possible that BNCs could serve a similar function for anti-neutrophil therapies. In clinical practice it is helpful to identify patients at higher risk of exacerbation or mortality in order to intensify therapy or provide closer monitoring. One potential biomarker that has been investigated is the neutrophil-lymphocyte ratio (NLR), however, the move from using relative to absolute eosinophil counts suggests that a ratio could be harder to apply clinically meaningful cutoffs to, whereas the use of BNC could be easier to interpret than the NLR [32]. Our study, which is an order of magnitude larger than the studies on NLR in COPD [33], suggests that patients with an elevated BNC when stable who do not have an obvious alternative cause of an increased BNC may be at higher risk of COPD complications and therefore may require closer observation.

Our study has important limitations. The TARDIS study was retrospective and has the limitations of observational research. It is older than other similar cohorts that have been examined, though neither adjusting the models for age nor restricting the analysis to the younger individuals changed the results. We used ICD codes for classification of mortality and these have well described limitations of accuracy. Nevertheless, our sample size and the consistency of our results across multiple subanalyses suggest no important bias. The use of oral corticosteroid prescription to identify exacerbations is well established in COPD studies using electronic health records but this approach has limitations. Our very large sample size and the results of sensitivity analyses including antibiotic prescriptions suggest exacerbation definition is unlikely to have had a major effect on the results. Although we used a conservative strategy to avoid capturing BNCs during acute infections, we were not able to prospectively identify the underlying reasons for elevated BNCs in these patients. The mechanistic sub-cohort study is limited by a relatively small sample size, despite being among the largest reported COPD microbiome studies, and so replication of our results in larger cohorts, other than ECLIPSE, would be valuable.

In summary, this large observational cohort study has identified BNCs as a strong and consistent predictor of future exacerbations and mortality in stable COPD. The neutrophil remains central to the pathophysiology of COPD and therapeutic development targeting neutrophilic inflammation in appropriate patients is urgently needed.

## Supplementary information

**Additional file 1.**

## Data Availability

Data are available from the Health Informatics Centre, University of Dundee, Ninewells Hospital, Dundee, DD1 9SY, UK.

## Abbreviations

95%CI: 95% Confidence interval
BMI: Body mass index
BNC: Blood neutrophil count
BODE: Body-mass index, airflow obstruction, dyspnea and exercise index
CAT: COPD assessment test
COPD: Chronic obstructive pulmonary disease
FEV_1_: Forced expiratory volume in 1 s
FVC: Forced vital capacity
GLM: Generalized linear model
HR: Hazard ratio
ICD10: International classification of diseases 10th revision
ICS: Inhaled corticosteroid
IQR: Interquartile range
MRC: Medical research council
NE: Neutrophil elastase
NET: Neutrophil extracellular trap
NLR: Neutrophil-Lymphocyte ratio
ROS: Reactive oxygen species
TARDIS: Tayside allergy and respiratory disease information system
SGRQ: St George’s respiratory questionnaire

## References

[1] Brightling C, Greening N. Airway inflammation in COPD- progress to precision medicine. Eur Respir J. 2019;54(2):1900651. 10.1183/13993003.00651-2019. 10.1183/13993003.00651-2019 31073084

[2] Cowburn AS, Condliffe AM, Farahi N, Summers C, Chilvers ER (2008). Advances in neutrophil biology: clinical implications. Chest.

[3] Stockley JA, Walton GM, Lord JM, Sapey E (2013). Aberrant neutrophil functions in stable chronic obstructive pulmonary disease: the neutrophil as an immunotherapeutic target. Int Immunopharmacol.

[4] Oliveira MV, Abreu SC, Padilha GA (2016). Characterization of a mouse model of emphysema induced by multiple instillations of low-dose Elastase. Front Physiol.

[5] Suki B, Bartolak-Suki E, Rocco PRM (1639). Elastase-induced lung emphysema models in mice. Methods Molecular Biol.

[6] Ghorani V, Boskabady MH, Khazdair MR, Kianmeher M (2017). Experimental animal models for COPD: a methodological review. Tob Induc Dis.

[7] Dicker AJ, Crichton ML, Pumphrey EG (2018). Neutrophil extracellular traps are associated with disease severity and microbiota diversity in patients with chronic obstructive pulmonary disease. J Allergy Clin Immunol.

[8] Cockayne DA, Cheng DT, Waschki B (2012). Systemic biomarkers of neutrophilic inflammation, tissue injury and repair in COPD patients with differing levels of disease severity. PLoS One.

[9] Wouters EF, Groenewegen KH, Dentener MA, Vernooy JH (2007). Systemic inflammation in chronic obstructive pulmonary disease: the role of exacerbations. Proc Am Thorac Soc.

[10] Dicker AJ, Crichton ML, Cassidy AJ (2018). Genetic mannose binding lectin deficiency is associated with airway microbiota diversity and reduced exacerbation frequency in COPD. Thorax..

[11] Vestbo J, Anderson W, Coxson HO (2008). Evaluation of COPD longitudinally to identify predictive surrogate end-points (ECLIPSE). Eur Respir J.

[12] Barnes PJ, Celli BR (2009). Systemic manifestations and comorbidities of COPD. Eur Respir J.

[13] Sinden NJ, Stockley RA (2010). Systemic inflammation and comorbidity in COPD: a result of 'overspill' of inflammatory mediators from the lungs? Review of the evidence. Thorax.

[14] Mayhew D, Devos N, Lambert C (2018). Longitudinal profiling of the lung microbiome in the AERIS study demonstrates repeatability of bacterial and eosinophilic COPD exacerbations. Thorax.

[15] Faner R, Sibila O, Agustí A (2017). The microbiome in respiratory medicine: current challenges and future perspectives. Eur Respir J.

[16] Brinkmann V, Reichard U, Goosmann C (2004). Neutrophil extracellular traps kill bacteria. Science.

[17] Hoenderdos K, Condliffe A (2013). The neutrophil in chronic obstructive pulmonary disease. Am J Respir Cell Mol Biol.

[18] Milara J, Juan G, Peiro T, Serrano A, Cortijo J (2012). Neutrophil activation in severe, early-onset COPD patients versus healthy non-smoker subjects in vitro: effects of antioxidant therapy. Respiration.

[19] Sapey E, Stockley JA, Greenwood H (2011). Behavioral and structural differences in migrating peripheral neutrophils from patients with chronic obstructive pulmonary disease. Am J Respir Crit Care Med.

[20] Hidalgo A, Chilvers ER, Summers C, Koenderman L. The neutrophil life cycle. Trends Immunol. 2019;40(7):584–97. 10.1016/j.it.2019.04.013. 10.1016/j.it.2019.04.013 31153737

[21] Jones AW, Robinson R, Mohamed P, Davison G, Izzat HJ, Lewis KE (2016). Impaired blood neutrophil function in the frequent Exacerbator of chronic obstructive pulmonary disease: a proof-of-concept study. Lung.

[22] Nussbaumer-Ochsner Y, Rabe KF (2011). Systemic manifestations of COPD. Chest.

[23] Agusti A, Edwards LD, Rennard SI (2012). Persistent systemic inflammation is associated with poor clinical outcomes in COPD: a novel phenotype. PLoS One.

[24] Hurst JR, Vestbo J, Anzueto A (2010). Susceptibility to exacerbation in chronic obstructive pulmonary disease. N Engl J Med.

[25] Perera WR, Hurst JR, Wilkinson TM (2007). Inflammatory changes, recovery and recurrence at COPD exacerbation. Eur Respir J.

[26] Patel IS, Vlahos I, Wilkinson TM (2004). Bronchiectasis, exacerbation indices, and inflammation in chronic obstructive pulmonary disease. Am J Respir Crit Care Med.

[27] Mannino DM, Thorn D, Swensen A, Holguin F (2008). Prevalence and outcomes of diabetes, hypertension and cardiovascular disease in COPD. Eur Respir J.

[28] Divo MJ, Casanova C, Marin JM (2015). COPD comorbidities network. Eur Respir J.

[29] Divo M, Cote C, de Torres JP (2012). Comorbidities and risk of mortality in patients with chronic obstructive pulmonary disease. Am J Respir Crit Care Med.

[30] Vogelmeier C, Aquino TO, O'Brien CD, Perrett J, Gunawardena KA (2012). A randomised, placebo-controlled, dose-finding study of AZD9668, an oral inhibitor of neutrophil elastase, in patients with chronic obstructive pulmonary disease treated with tiotropium. COPD.

[31] Lazaar AL, Miller BE, Tabberer M, et al. Effect of the CXCR2 antagonist danirixin on symptoms and health status in COPD. Eur Respir J. 2018;52(4):1801020. 10.1183/13993003.01020-2018. 10.1183/13993003.01020-2018 30139779

[32] Singh D, Agusti A, Anzueto A, et al. Global strategy for the diagnosis, management, and prevention of chronic obstructive lung disease: the GOLD science committee report 2019. Eur Respir J. 2019;53(5):1900164. 10.1183/13993003.00164-2019. 10.1183/13993003.00164-2019 30846476

[33] Paliogiannis P, Fois AG, Sotgia S (2018). Neutrophil to lymphocyte ratio and clinical outcomes in COPD: recent evidence and future perspectives. Eur Respir Rev.

