# Supplementary material for: Blood neutrophil counts are associated with exacerbation frequency and mortality in COPD
Source: Respir Res. 2020 Jul 1;21:166. doi: 10.1186/s12931-020-01436-7 (PMC7329438; doi:10.1186/s12931-020-01436-7)
Supplement: Supplementary file 1 — Additional file 1. [file 12931_2020_1436_MOESM1_ESM.docx]

Online Supplement For: **Blood Neutrophil Counts are Associated with Exacerbation Frequency and Mortality in COPD**

Mike Lonergan*^1^, Alison J Dicker*^1^, Megan L Crichton^1^, Holly R Keir, Melissa K. Van Dyke^2^, Hana Mullerova^3^, Bruce E Miller^4^, Ruth Tal-Singer^4^, James D Chalmers^1^

*Joint first authors

^1^Scottish Centre for Respiratory Research, University of Dundee, United Kingdom

^2^Epidemiology, Value Evidence and Outcomes GSK R&D, Collegeville, PA, USA

^3^ Epidemiology, Value Evidence and Outcomes, GSK R&D, Uxbridge, UK

^4^Medical Innovation, Value Evidence and Outcomes, GSK R&D, Collegeville, PA, USA^2^

Corresponding author: James D Chalmers, Scottish Centre for Respiratory Research, University of Dundee, Ninewells Hospital and Medical School, Dundee, DD1 9SY, [jchalmers@dundee.ac.uk](mailto:jchalmers@dundee.ac.uk)

**Supplementary Methods**

**Microbiome Sequencing**

DNA was extracted from a minimum of 0.1 g whole induced sputum. 16S rRNA gene sequencing was performed using the Illumina library prep guide (https://www.illumina.com/content/dam/illumina-support/documents/documentation/chemistry_documentation/16s/16s-metagenomic-library-prep-guide-15044223-b.pdf), using primers targeting the V3 and V4 region.^1^ Multiplexing of samples was achieved with Nextera XT Indices and the libraries sequenced using 2 x 300 paired end sequencing on the MiSeq platform using a MiSeq V3 kit (Illumina). Extraction, amplification and sequencing of matched water negative controls was performed to act as contamination controls. FastQ files were imported into QIIME (version 1.9.0) and paired end reads were joined together for each sample and the quality of reads checked; any reads with a Phred quality score less than Q20 were excluded. Un-joined reads were excluded from subsequent analysis. Sequences were clustered into operational taxonomic units (OTUs) based on 97% sequence similarity using the UCLUST algorithm,^2^ aligned against the Greengenes Core reference alignment (Version 13.8)^3^ using PyNAST (Version 1.2.2).^4^ Taxonomy of the OTUs was assigned using the Ribosomal Database Project Classifier (Version 2.2) with the *de novo* OTU picking option.^5^ OTUs were filtered to remove singletons and unassigned OTUs, or OTUs identified as Human, Eukaryota or Cyanobacteria. The dataset was normalized to a number of OTUs above those observed in the majority of negative controls, no sample passing quality checks had fewer reads than its related negative control, and the phylogeny of all the OTUs determined. Negative controls OTUs were compared to samples to identify potential contaminants; the numbers of sequence reads obtained from negative controls was markedly lower than samples. Shannon-Wiener Diversity Index (SWDI) and Dominance were calculated in PAST3 to determine alpha diversity of each sample. The dominant phyla or genera of individual samples was defined as the phyla or genus with the highest percentage.

| **Abbreviation** | **Description** |
| --- | --- |
| Index date | Index date, expressed as number of days elapsed since 1st January 1900. |
| Birth year | Year of birth |
| Height | Height – the last height (in m) recorded for the individual before their index date. |
| Weight | Weight – the last weight (in kg) recorded for the individual before their index date. |
| BMI | Body Mass Index: weight/ height^2; using last height and weight before index date (above) |
| SCSIMD5 | Socioeconomic status –this is the quintile (1= most deprived, 5 = most affluent) of the Scottish index of multiple deprivation for the individual’s last recorded postcode |
| Exacerbations | Number of acute exacerbations in the year up to index date. These were defined as distinct occasions when the individual received a short course, either in the community or in A&E, of prednisolone (150mg< total dose < 1g; if daily dose recorded it must be >30mg, though directions are only available for ~30% of community prescriptions). (Inpatient prescriptions are not recorded in the TARDIS database.) |
| Severe exac. | Number of severe exacerbations in the year up to index date. These were defined as hospital inpatient admissions where the main condition was recorded as being within ICD10 code J44. |
| GOLD | GOLD score (A-D) based on acute exacerbations, severe exacerbations and MRC dyspnoea scores recorded in the year up to index date |
| Eosinophils | The last blood eosinophil count recorded for the individual in the year up to their index date. This is almost always from the same date as the BNC. |
| ICS | Received at least one prescription for inhaled corticosteriods (budesonide, ciclesonide, fluticasone, beclometasone, mometasone) in the year up to the index date. |
| LABA | Received at least one prescription for long acting beta2-agonists (olodaterol, indacaterol, formoterol, salmeterol, vilanterol) in the year up to the index date. |
| LAMA | Received at least one prescription for long-acting muscarinic antagonists (aclidinium, tiotropium, glycopyrronium, umeclidinium) in the year up to the index date. |
| SABA | Received at least one prescription for short acting beta2-agonists (salbutamol, terbutaline) in the year up to the index date. |
| SAMA | Received at least one prescription for short-acting muscarinic antagonists (ipratropium) in the year up to the index date. |
| Macrolides | Received at least one prescription for macrolides (erythromycin, clarithromycin, azithromycin) in the year up to the index date. |
| Mucolytics | Received at least one prescription for mucolytics (carbocisteine, erdosteine) in the year up to the index date. |
| Theophylline | Received at least one prescription for theophylline in the year up to the index date. |
| Antibiotics | Received at least one prescription for relevant antibiotics (amoxicillin; co-amoxiclav; doxycycline; clarithromycin) in the year up to the index date. |
| Short ab | The number of short courses (prescriptions lasting 5-7 days, where directions were recorded) of antibiotics (for a total amount of: up to 21g total amoxicillin; 9-14g co-amoxiclav; up to 1.4g doxycycline; or up to 7g clarithromycin) in the year up to the index date. |
| MITT1 | Multiple inhaler triple therapy. Definition 1: At least 1 day in the year up to the index date where the coverage of a LABA a LAMA and an ICS prescription overlapped. |
| MITT2 | Multiple inhaler triple therapy. Definition 2: At least 2 prescriptions of LABA, LAMA and ICS received in the year up to index date. |
| Chronicbronchitis | Either a hospital inpatient admission where ICD10 code J41 or J42 is recorded as the main condition in the year up to the index date, or a record of sputum production at a TARDIS visit. |
| CMHs | Chronic mucus hypersecretion, defined as more than one record of both cough and sputum production, within the TARDIS dataset, in the year up to the index date. |
| Asthma | A record in TARDIS or EMBARC of the individual reporting having asthma, or an admission with main condition recorded as ICD10 code J10 before the individuals’ index date. |
| Cancer | A diagnosis of any cancer recorded the Scottish Cancer Registry. TARDIS or EMBARC. |
| CVD | Any record of cardiovascular disease in the EMBARC or TARDIS datasets or an admission, with main condition in ICD codes I20-I25 in the year up to index date. |
| Depressionp | Depression: At least one prescription within BNF code 4.3 in the year to index date. |
| Diabetes | Diabetes: either presence in the SCIDIABETES dataset before the index date or a prescription within BNF code 6.1in the year to index, or diabetes recorded in the EMBARC database. |
| Hypertension | Hypertension: a hospital inpatient admission where ICD10 code I1 is recorded as the main condition in the year up to the index date |
| Osteoporosis | Either a record of osteoporosis in the TARDIS or EMBARC datasets or receipt of a prescription within BNF code 6.6. |
| Stroke | Either a hospital inpatient admission where the main condition was recorded as being within ICD10 code I6 in the year up to the index date, or a record of “VascularComorbiditiesStrokeorTransientIschaemicAttack” in the EMBARC dataset. |
| ACEI | At least one prescription for an ACE inhibitor (BNF code 2.5.5.1) in the year to index date. |
| PPI | At least one prescription for proton pump inhibitors (BNF code 1.3.5) in the year to index date. |
| Statin | At least one prescription for a statin (BNF code in 2.12) in the year to index date. |

e-**Table 1:** Details of the confounding variables added into the Generalised Linear Models investigating the association between exacerbation rate and blood neutrophil counts. Two different definitions of multiple inhaler triple therapy were included.


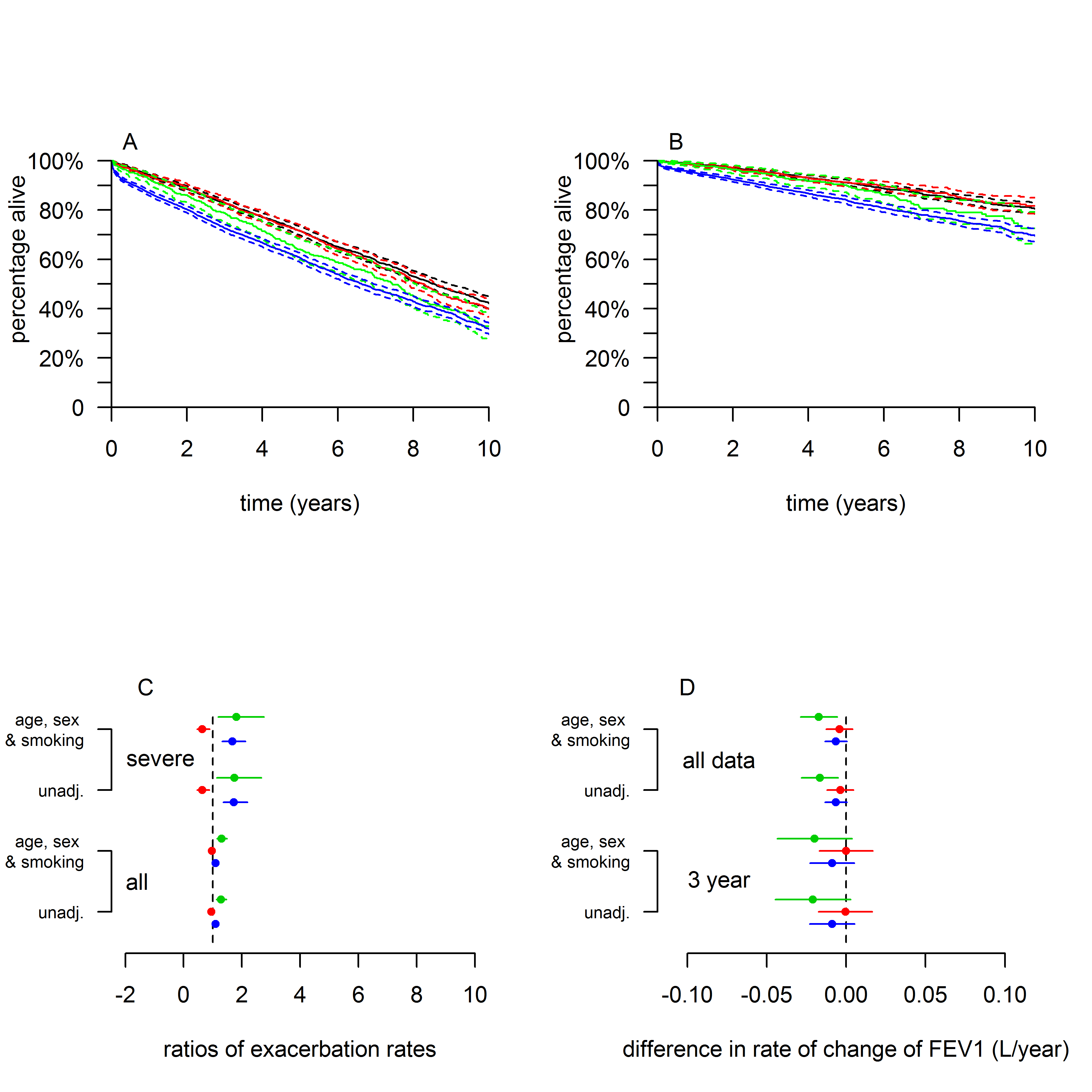


**e-Figure 1:** **A**: Kaplan Meier survival curves, with 95% confidence intervals, showing all-cause mortality for the blood eosinophil groups adjusted for age, sex, and smoking status. The low eosinophil group is blue; the normal group is black; the elevated group is red and the extreme group is green. **B**: As **A**, but representing mortality recorded as ICD10 J44, with all other mortality as a competing risk. **C**: Ratios of the numbers of all, and severe, exacerbations over the year from the index date from both unadjusted models and models adjusted for age, sex and smoking status. All are relative to the normal blood eosinophil group. **D:** Rates of change of FEV_1_, estimated by fitting mixed models to either all data or only that obtained in the three years from each individual’s index date.

| Response | |  | unadjusted | adjusted for age, sex and smoking |
| --- | --- | --- | --- | --- |
| Mortality | all | hazard ratio | 1.06 (0.96, 1.16) | 1.03 (0.94, 1.13) |
|  | J44 |  | 0.96 (0.79, 1.16) | 0.95 (0.78, 1.15) |
| Exacerbations | all | rate ratio | -0.049  (-0.147, 0.049) | -0.031  (-0.129, 0.067) |
|  | severe |  | -0.45  (-0.78, -0.11) | -0.44  (-0.78, -0.11) |
| FEV1 decline | all data | L/year | -0.0037  (-0.0122 0.0048) | -0.0042  (-0.0127, 0.0043) |
|  | 3 year |  | -0.0005  (-0.0177, 0.0168) | -0.0001  (-0.0173, 0.0171) |

**e-Table 2**: Effect sizes (95% confidence intervals), for the group with elevated blood eosinophils relative to those with eosinophils in the normal range, estimated from the models.


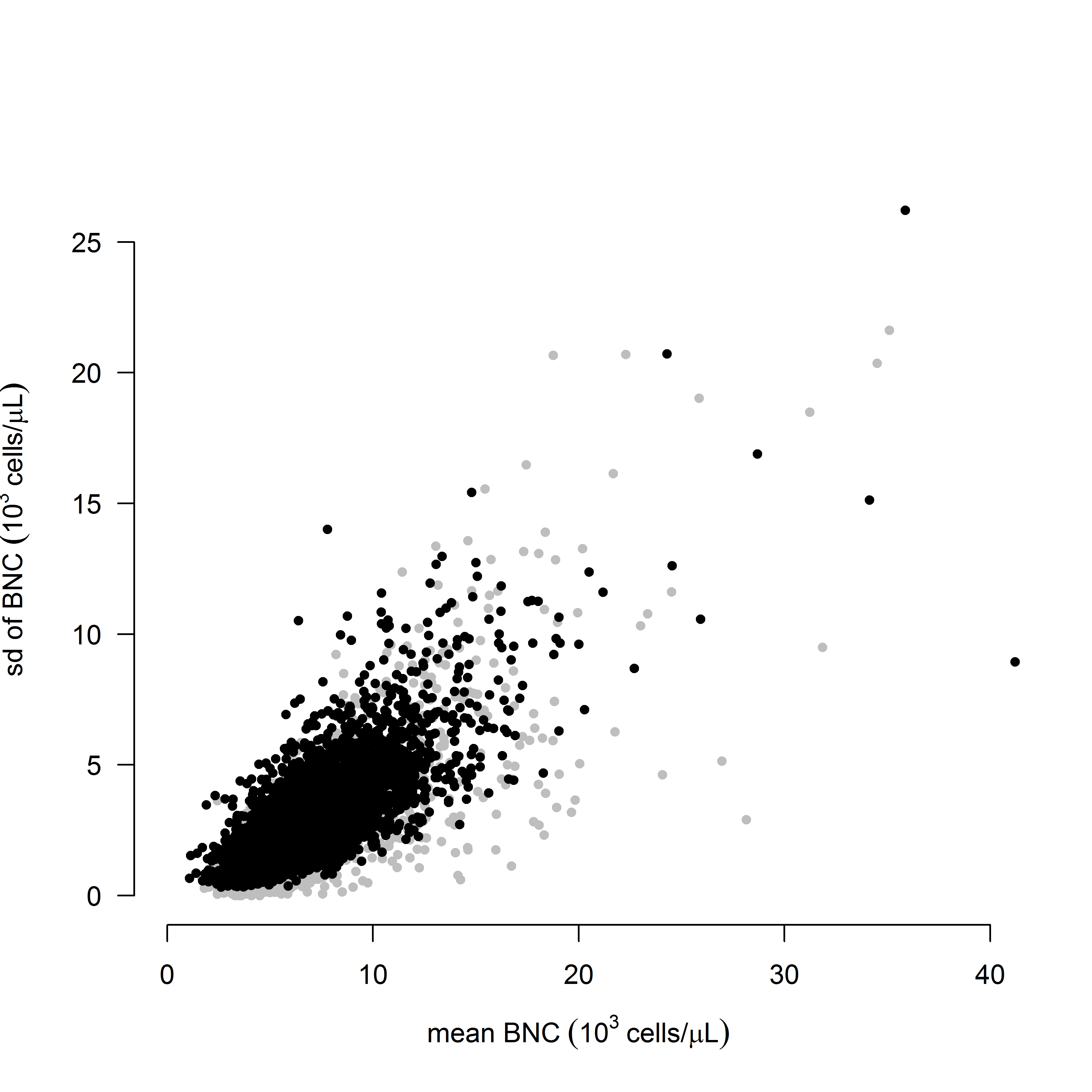


**e-Figure 2:** The mean and standard deviation of stable BNC for each individual, those with ten or less measurements are shown in grey.


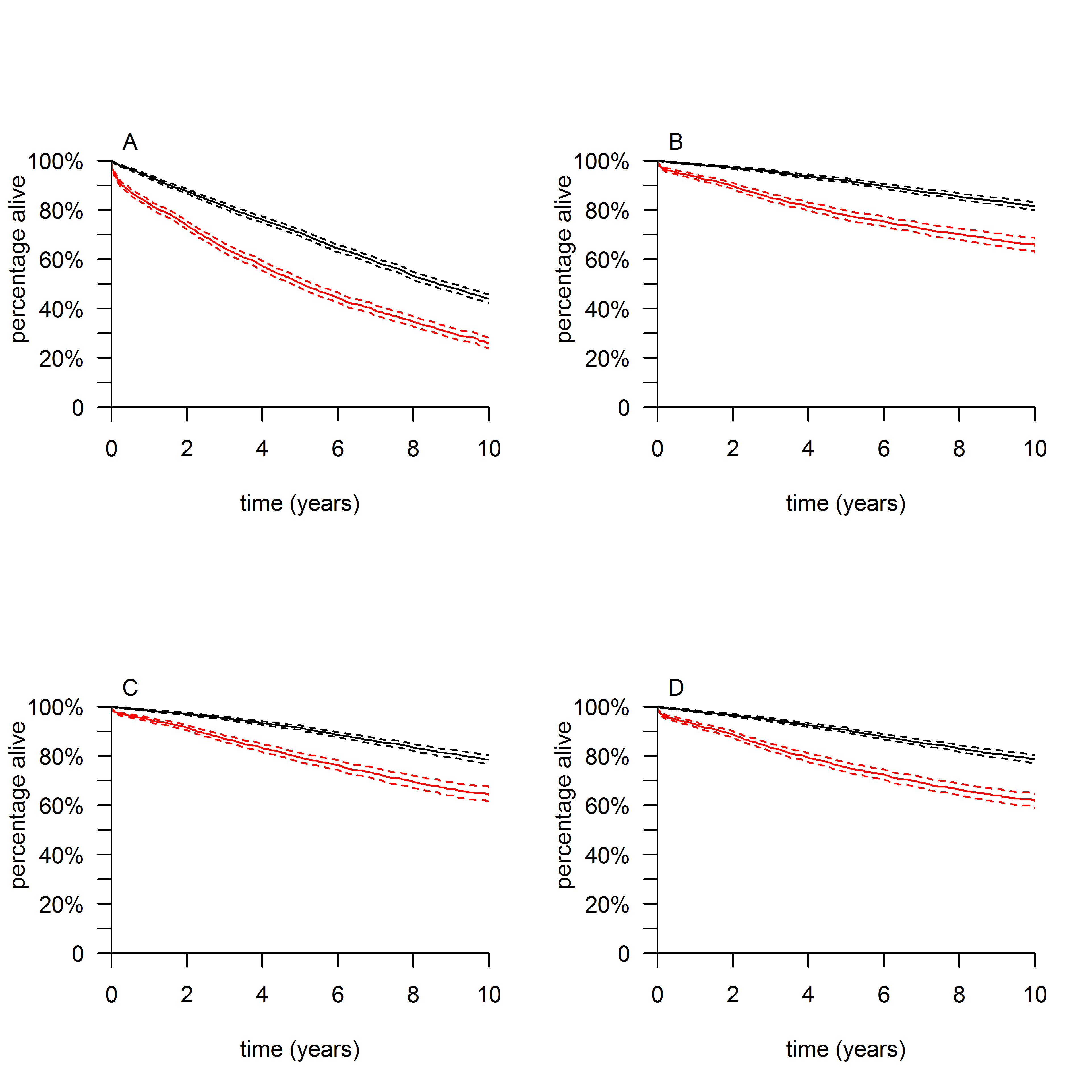


**e-Figure 3**: Kaplan-Meier survival curves for: **A:** all mortality, unadjusted model; **B:** mortality from J44, unadjusted model; **C:** mortality from all ICD J codes; model adjusted for age sex and smoking status; **D**: as **C**, but unadjusted model.

| Response | |  | unadjusted | adjusted for age, sex and smoking |
| --- | --- | --- | --- | --- |
| Mortality | all | hazard ratio | 1.80 (1.68, 1.91) | 1.67 (1.57, 1.78) |
|  | J44 |  | 2.45 (2.17, 2.76) | 2.27 (2.01, 2.56) |
| Exacerbations | all | rate ratio | 0.323  0.254, 0.392) | 0.334  0.265, 0.403) |
|  | severe |  | 1.00  0.79, 1.22) | 1.01  (0.79,1.23) |
| FEV1 decline | all data | L/year | -0.0012  -0.0017, -0.0008) | -0.0010  -0.0014, -0.0006) |
|  | 3 year |  | -0.0013 (  -0.0018, -0.008) | -0.0011  ( -0.0015, -0.0007) |

**e-Table 3**: Effect sizes (95% confidence intervals), for the group with elevated blood neutrophils relative to those with neutrophils in the normal range, estimated from the models.

**
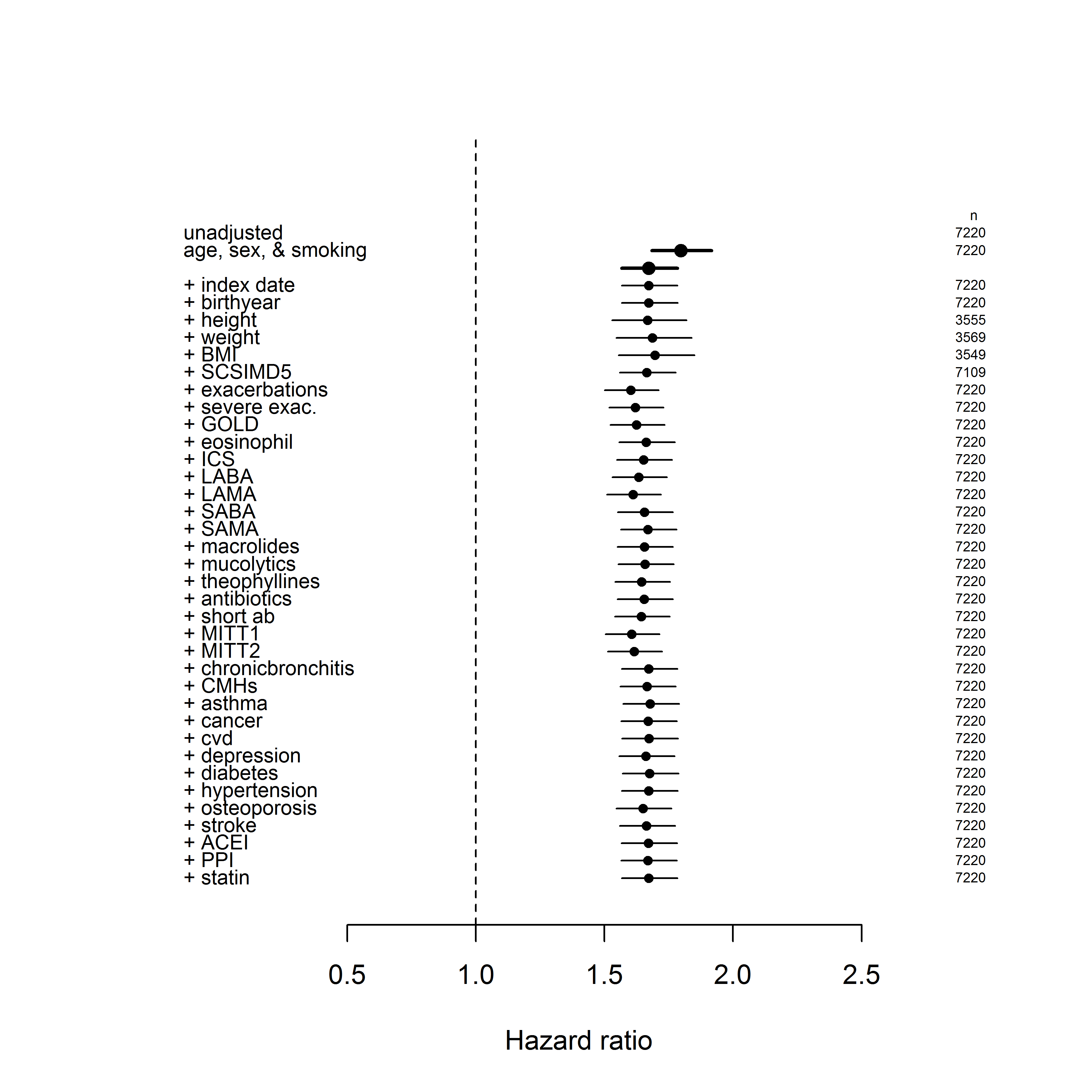
e-Figure 4:** Hazard rates comparing all-cause mortality in the elevated and normal BNC groups. Each line is a point estimate and 95% confidence interval for one model. Below the unadjusted result, and that adjusted for age, sex, and smoking, the other results are each for adding one term to the adjusted model. The labels on the left correspond to e-Table 1, and the numbers on the right are sample sizes.


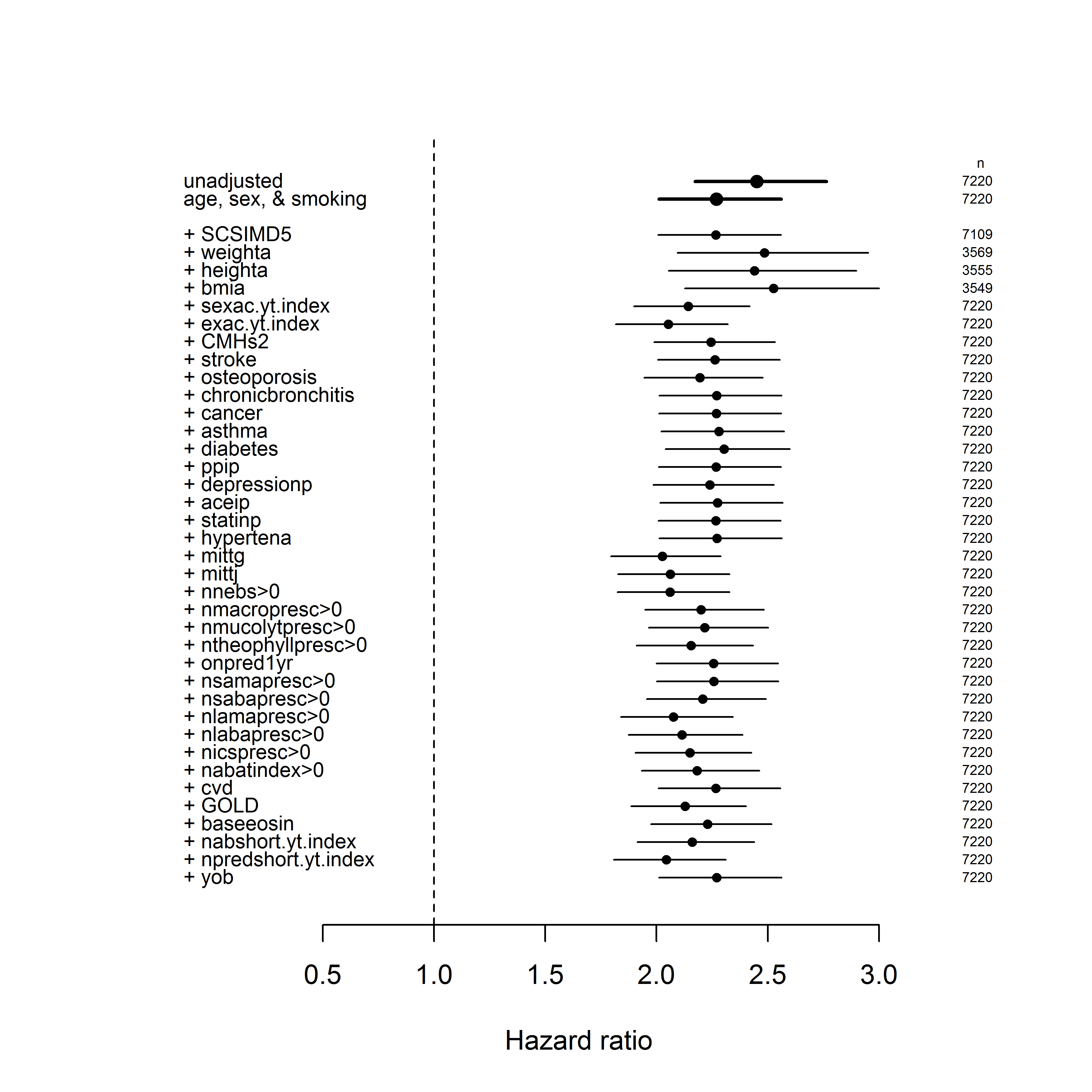
**e-Figure 5**: as e-Figure 4, but for only mortality from ICD10 code J44. Each line is a point estimate and 95% confidence interval for one model. Below the unadjusted result, and that adjusted for age, sex, and smoking, the other results are each for adding one term to the adjusted model. The labels on the left correspond to e-Table 1, and the numbers on the right are sample sizes.

**
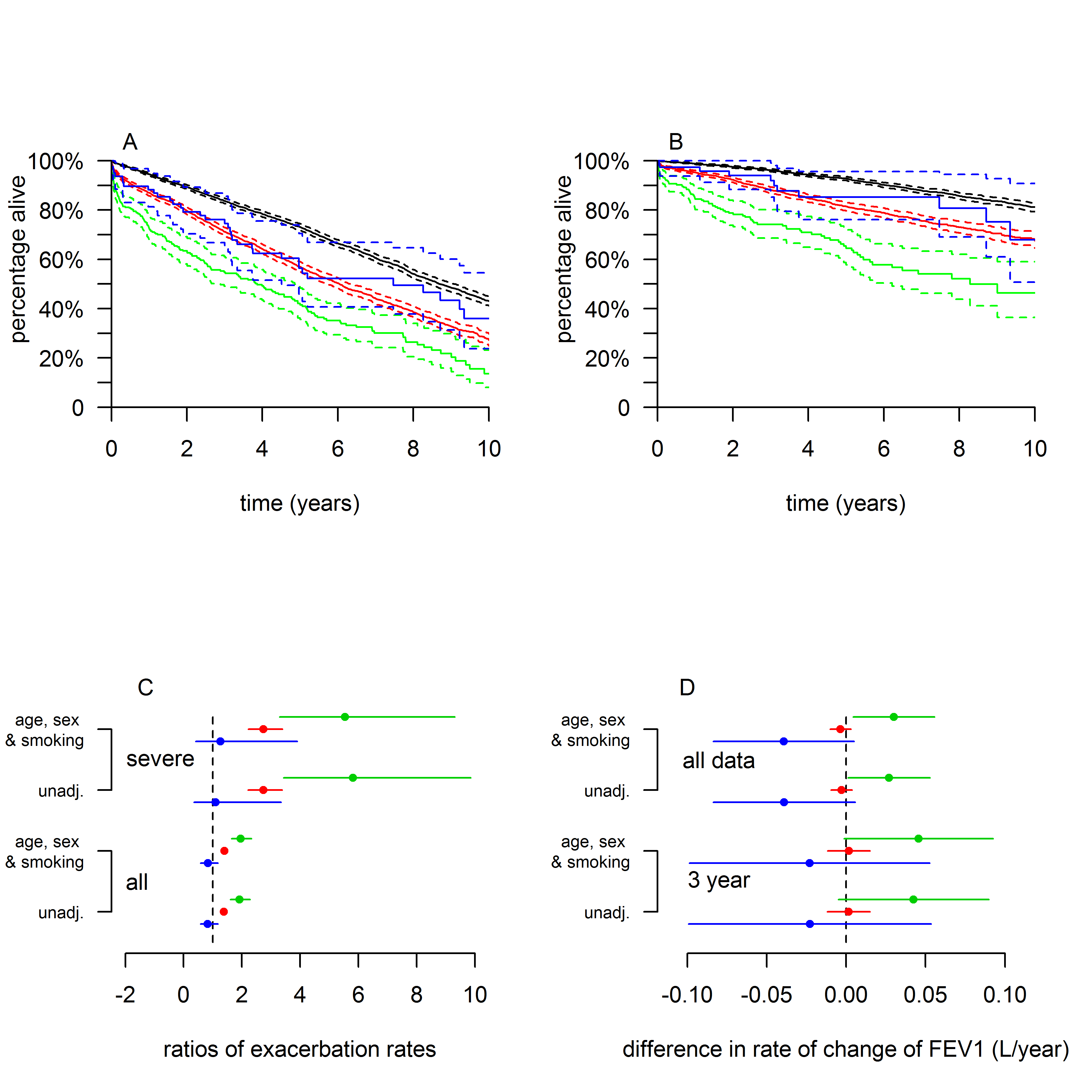
e-Figure 6**: **A**: Kaplan Meier survival curves, with 95% confidence intervals, showing low neutrophil group (blue) and extreme neutrophil group (green) alongside the normal (black) and elevated (red) groups. adjusted for age, sex, and smoking status. **B**: As **A**, but representing mortality recorded as ICD10 J44, with all other mortality as a competing risk. **C**: Ratios of the numbers of all, and severe, exacerbations over the year from the index date from both unadjusted models and models adjusted for age, sex and smoking status. All are relative to the normal BNC group. **D:** Rates of change of FEV_1_, estimated by fitting mixed models to either all data or only that obtained in the three years from each individual’s index date. Note that the x-axes in **C** and **D** have been extended to show the extra data compared to that shown in Figure 3.
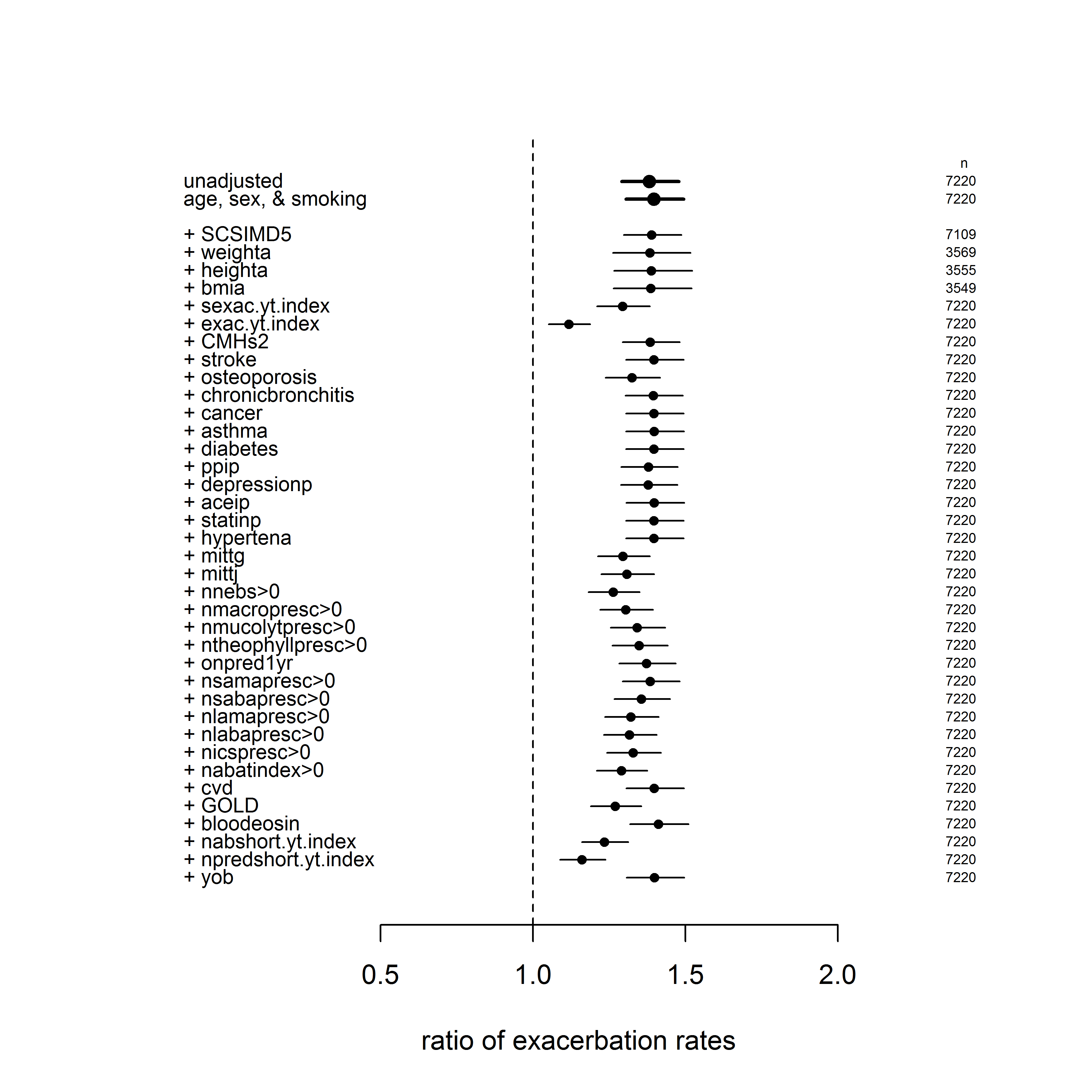
**e-Figure 7:** Incident rate ratios, compared to the normal BNC group, for all exacerbations for elevated BNC groups. Each row shows the mean and 95% confidence interval for the model adjusted for one of 35 potential confounding variables, listed in e-Table 1.
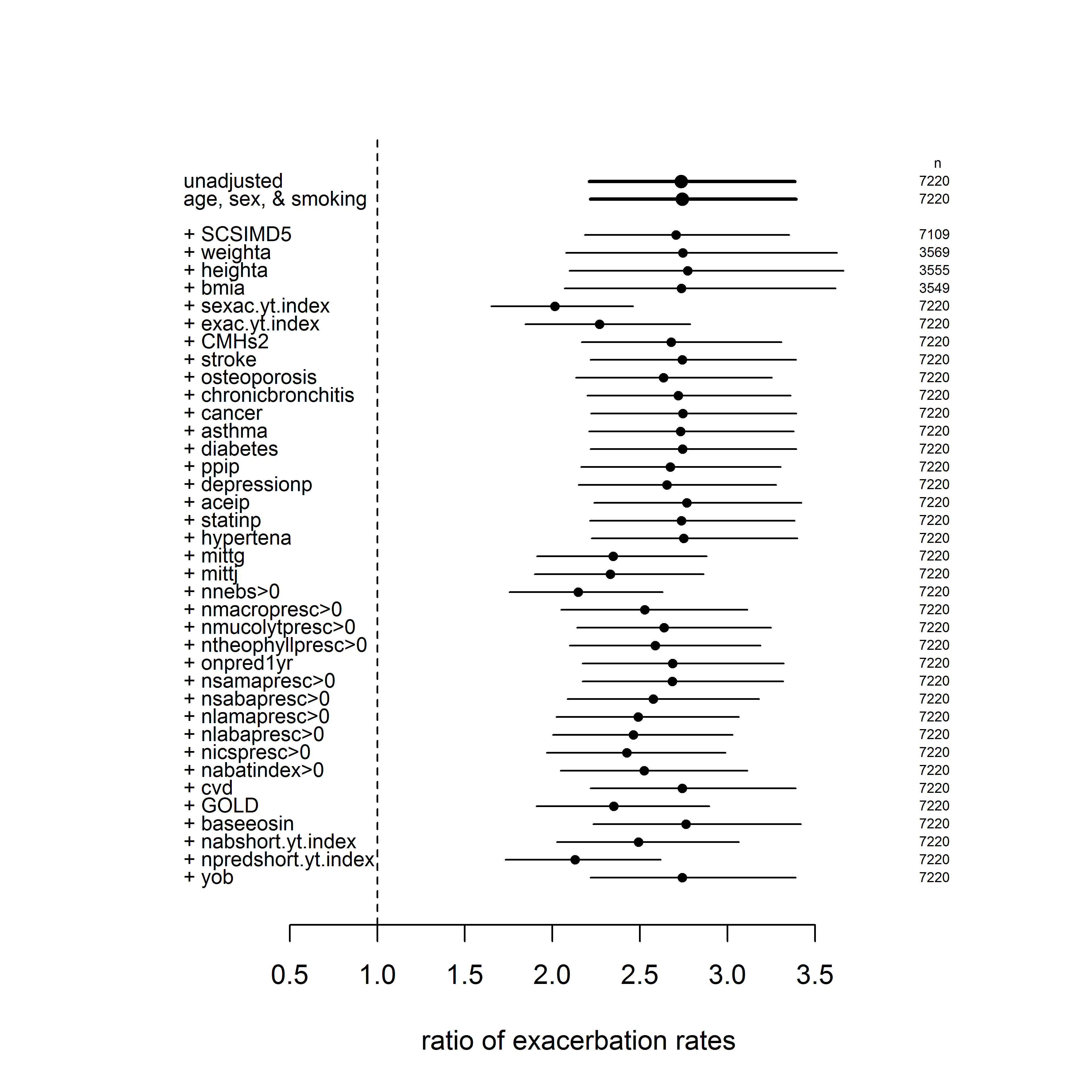
**e-Figure 8:** as e-Figure 7, but for only severe exacerbations, those causing hospitalization. Each row shows the mean and 95% confidence interval for the model adjusted for one of 38 potential confounding variables, listed in e-Table 1.


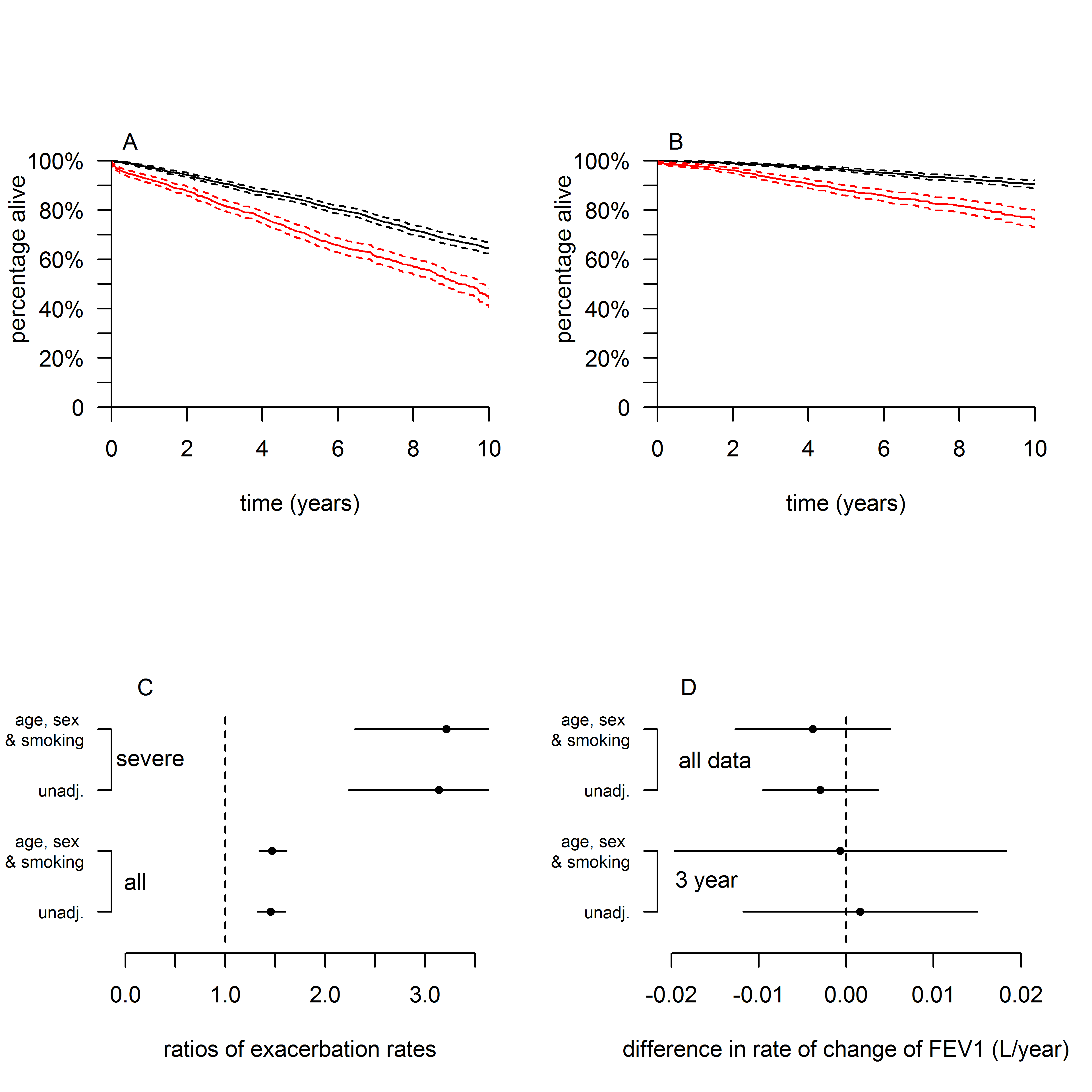


**e-Figure 9**: As Figure 3 but restricted to the younger half (age<74 at index date) of the cohort.


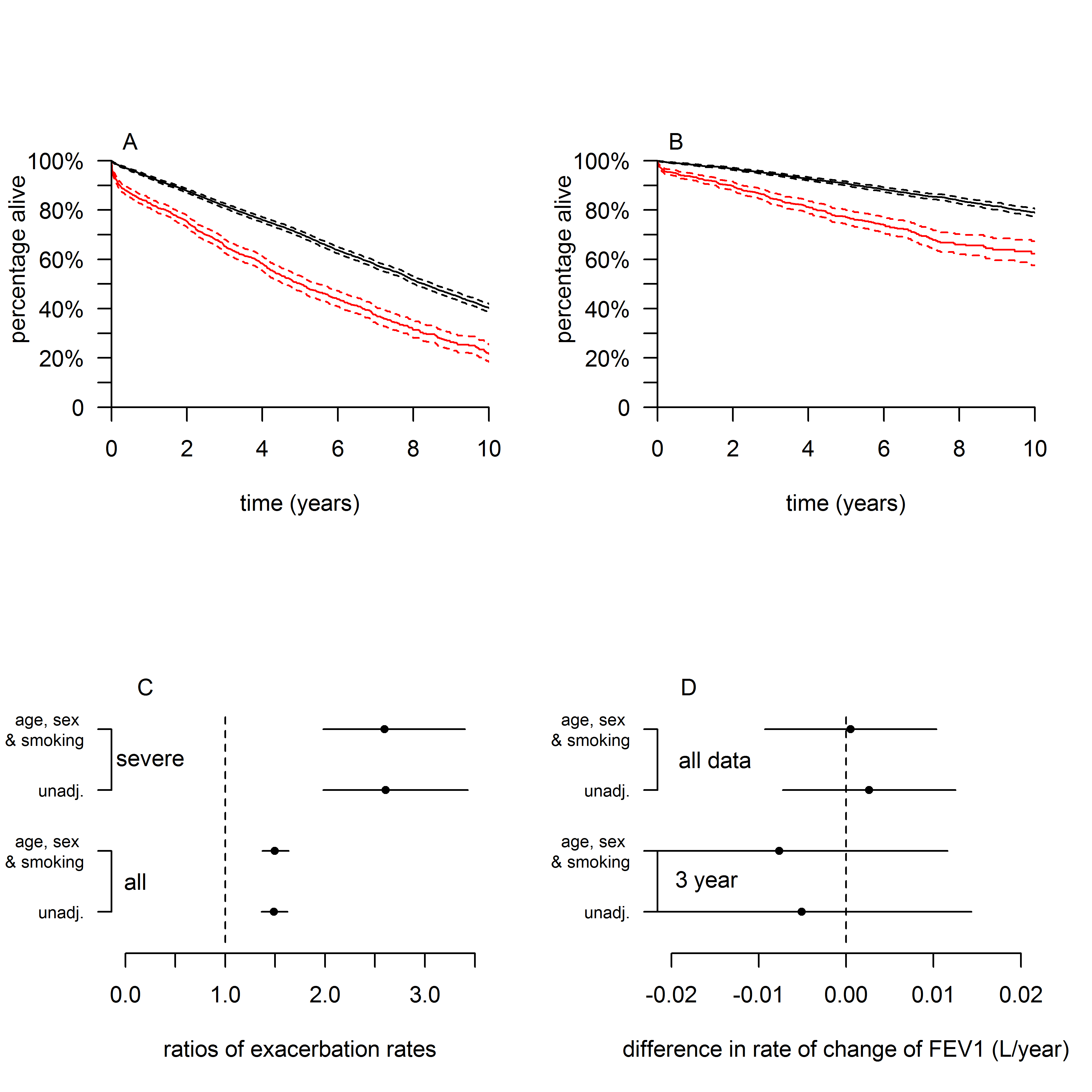


**e-figure 10**: As Figure 3, but with the elevated BNC group defined as 8,000-15,000 cells/µL, rather than 6,000-15,000 cells/µL.

|  | **Median (IQR) or n (%)** |
| --- | --- |
| Age | 71 (66 -78) |
| Male Gender | 166 (60.81) |
| BMI | 26.52 (24.00 -31.00) |
| Smoking status  Never  Ex  Current | 4 (1.47)  196 (71.79)  73 (26.74) |
| ICS use* | 176 (64.47) |
| MRC dyspnoea score | 3 (2 -4) |
| Exacerbation frequency (year prior to the study)  0  1  2  3 or more  Severe exacerbation requiring hospitalization (year prior to the study) | 61 (22.34)  53 (19.41)  40 (14.65)  119 (43.59)  63 (23.08) |
| Daily Sputum volume (mL) | 10 (3.00 -20.00) |
| Spirometry  FEV_1_ %  FVC (L)  FEV_1_/FVC  ≥80  50-79  30-49  <30 | 65 (50.43 -80)  2.90 (2.19 -3.61)  3 (1.10)  158 (57.88)  101 (37.00)  11 (4.03) |
| GOLD 2017  A  B  C  D | 67 (24.54)  36 (13.19)  53 (19.41)  117 (42.86) |
| SGRQ total score  CAT total score | 47.82 (31.08 -64.50)  19 (14 -25) |

e-**Table 4:** Characteristics of the mechanistic subcohort (n=273 unique subjects, all assessed when clinically stable). Abbreviations: ICS= inhaled corticosteroids, BMI= body mass index, MRC= Medical Research Council, FEV_1_= forced expiratory volume in 1 second, FVC= forced vital capacity, SGRQ= St Georges Respiratory Questionnaire, CAT= COPD assessment test. *includes medication used in combination with other bronchodilators or inhaled steroids.





e-Figure 11: Stacked microbiome profiles of the 246 patients in the mechanistic sub-cohort, shown at the **A:** Phylum and **B:** Genera level. Each vertical bar represents one stable patient, grouped according to their BNC. The one patient with a low BNC (<2000cells/µL) is on the left (*) whilst patients with an extreme BNC (>15000cells/µL) are on the right (#).

**Supplementary references:**

1. Klindworth A, Pruesse E, Schweer T, et al. Evaluation of general 16S ribosomal RNA gene PCR primers for classical and next-generation sequencing-based diversity studies. *Nucleic Acids Res.* 2013;41(1).

2. Edgar RC. Search and clustering orders of magnitude faster than BLAST. *Bioinformatics.* 2010;26(19):2460-2461.

3. DeSantis TZ, Hugenholtz P, Larsen N, et al. Greengenes, a chimera-checked 16S rRNA gene database and workbench compatible with ARB. *Appl Environ Microbiol.* 2006;72(7):5069-5072.

4. Caporaso JG, Bittinger K, Bushman FD, DeSantis TZ, Andersen GL, Knight R. PyNAST: a flexible tool for aligning sequences to a template alignment. *Bioinformatics.* 2010;26(2):266-267.

5. Wang Q, Garrity GM, Tiedje JM, Cole JR. Naive Bayesian classifier for rapid assignment of rRNA sequences into the new bacterial taxonomy. *Appl Environ Microbiol.* 2007;73(16):5261-5267.
